# Correcting duplicate publications: follow up study of MEDLINE tagged duplications

**DOI:** 10.11613/BM.2019.010201

**Published:** 2018-12-15

**Authors:** Mario Malički, Ana Utrobičić, Ana Marušić

**Affiliations:** 1 Department of Research in Biomedicine and Health, University of Split School of Medicine, Split, Croatia; 2 Department of Medical Humanities, University of Split School of Medicine, Split, Croatia; 3 Central Medical Library, University of Split School of Medicine, Split, Croatia

**Keywords:** duplicate publication, retraction of publication, authorship, scientific misconduct, published erratum

## Abstract

**Introduction:**

As MEDLINE indexers tag similar articles as duplicates even when journals have not addressed the duplication(s), we sought to determine the reasons behind the tagged duplications, and if the journals had undertaken or had planned to undertake any actions to address them.

**Materials and methods:**

On 16 January 2013, we extracted all tagged duplicate publications (DPs), analysed published notices, and then contacted MEDLINE and editors regarding cases unaddressed by notices. For non-respondents, we compared full text of the articles. We followed up the study for the next 5 years to see if any changes occurred.

**Results:**

We found 1011 indexed DPs, which represented 555 possible DP cases (in MEDLINE, both the original and the duplicate are assigned a DP tag). Six cases were excluded as we could not obtain their full text. Additional 190 (35%) cases were incorrectly tagged as DPs. Of 359 actual cases of DPs, 200 (54%) were due to publishers’ actions (*e.g*. identical publications in the same journal), and 159 (46%) due to authors’ actions (*e.g*. article submission to more than one journal). Of the 359 cases, 185 (52%) were addressed by notices, but only 25 (7%) retracted. Following our notifications, MEDLINE corrected 138 (73%) incorrectly tagged cases, and editors retracted 8 articles.

**Conclusions:**

Despite clear policies on how to handle DPs, just half (54%) of the DPs in MEDLINE were addressed by journals and only 9% retracted. Publishers, editors, and indexers need to develop and implement standards for better correction of duplicate published records.

## Introduction

Duplicate publication (DP) is a publication that substantially overlaps with one already published, but does not appropriately acknowledge its source (*1*). This practice can inflate an author’s or journal’s prestige, but wastes time and resources of readers, peer reviewers, and publishers (*2*, *3*). Duplication of data can also lead to biased estimates of efficacy or safety of treatments and products in meta-analyses of health interventions, as the same data which is calculated twice exaggerates the accuracy of the analysis, and leaves an impression that more patients were involved in testing a drug (*4*, *5*). Not referencing the origin or the overlap of the data, can therefore be considered akin to fabrication, as it implies the data or information is new, when in fact it is not. For these reasons, all major international editorial organizations, including the Committee on Publication Ethics (COPE), the Council of Science Editors (CSE), and the International Committee of Medical Journal Editors (ICMJE), recommend retracting DPs (*1*, *6*, *7*). A 2014 study of MEDLINE retractions showed that duplication accounted for 22% (506 out of 2343) of retracted publications 1 (*8*). Retracted publications in MEDLINE are tagged following the formal issuance of retraction by an authorized party (*e.g.* author or a journal). Duplicate publications, however, are tagged whenever substantial overlap between two or more articles is discovered during indexing, irrespective of an authorized notification (*9*, *10*). Furthermore, the DP [Publication Type] tag is given to both the duplicate(s) and to the original article(s). Indexers, however, do not routinely examine articles for originality, so the tagged DPs do not necessarily include all instances of DPs in MEDLINE (*10*).

The aim of our study was to investigate DPs indexed in MEDLINE, specifically whether they were retracted or corrected by journals, how visible those corrections were on the journals’ websites, and whether there was a change in DP citation counts after the publication of notices of duplication. Following the presentation of our initial findings at the Congress on Peer Review and Biomedical Publication in September 2013, we contacted National Library of Medicine (NLM) regarding possible indexing errors, and journal editors regarding unaddressed suspected duplications. Following their replies, for still unresolved cases we compared the full texts of DPs to confirm if they were indeed duplicate.

## Materials and methods

On 16 January 2013, we extracted all citations indexed as *duplicate publications* [Publication Type] in PubMed and created a database using the PubMed2XL^©^ software (*11*). We matched all extracted citations (N = 1011) by analysing the similarity in their titles, abstracts and authors in order to establish the number of cases of possible DPs. This revealed that among the 1011 citations extracted, there were 9 notices of duplicate publications and 1 letter to the editor that were (incorrectly) tagged as NLM tags notices of duplicate publications as a [Publication Type] Comment and with a Medical Subject Heading (MeSH) term *Duplicate publication as a topic*. Of 9 notices incorrectly tagged, 6 mentioned cases whose manuscripts were not tagged with the DP tag in MEDLINE, while 3 referred to cases whose manuscripts were also tagged. The letter to the editor described a case in which the authors realized during article proofing that a part of the study was already published and alerted the editors about it (*12*).

Of the remaining 1001 citations, 145 were citations with no similar articles tagged as DPs, while 856 could be matched with others and amounted to 400 possible cases of DPs. To determine if all were indeed DP cases, we investigated whether they had been acknowledged by the journal(s), by checking all linkages to the articles visible in MEDLINE or on the article’s website and obtaining the full texts of found notices. We then checked whether notices included the full title of the duplicate article in their heading, listed the reason behind the duplication, and were visible and freely accessible on the journal’s website, as recommended by the ICMJE and COPE (*1*, *6*). We also checked if the notices mentioned contacting the authors or included the authors’ explanation for the duplication. For the purpose of this study, the article that was published first was designated as original and the subsequent publication(s) as duplicate(s), unless otherwise stated in the published notice(s). Additionally, we classified the DPs as retracted if the notice was titled as retraction, or its wording mentioned the article was withdrawn, retracted or removed. Based on MEDLINE or journal online record we then extracted the following data: 1) the time (in months) from the original publication to duplicate publication, 2) the time (in months) from the duplicate publication to the publication of the notice, and 3) the difference in author by-line order between the original and duplicate publication. We also checked the citation records of DPs in the Web of Science (WoS) in May 2014. As *per* previous research, to allow for publication processing time and visibility of notices to reach the research community, we also separately collected citations that occurred two years after the publication of a notice (*13*).

Additionally, we informed NLM of possible indexing errors and contacted journal editors on 6-8 May 2015 regarding suspected unaddressed duplications for 250 possible cases of DPs, as no emails were available for 16 cases (template letter to the editors is available in Supplementary material). We sent personalised emails to editors and asked them if they were aware that the articles were tagged as DPs or that NLM was tagging publications as duplicate irrespective of official notices by journal(s). We received replies for 181 of 250 cases (72%).

For cases we did not receive the reply (following two reminders on 1-2 September and 28 October 2015, N = 69); for cases editors said they would investigate and forward us their findings, but never did so, even a year after we contacted them (N = 28); for cases editors did not specify what they would do (N = 7); for cases where editors said the other journal had a publication with a later date so they should investigate (N = 5), as well as for cases we couldn’t contact the editors by e-mail (N = 16) as journals ceased publishing or no contact was listed, we obtained the full text of the articles in question, and determined if they were indeed duplicates by manually comparing their text similarity and possible intentional republication (declaration or citation of the original article). For 6 cases we were unable to obtain the full text versions of articles or obtain the answers from editors, and so we excluded them from the analysis. In total, we manually compared 119 cases of possible DPs. Finally, we rechecked during May 2017 and again in June 2018, if the editors or NLM took any further actions regarding the cases we alerted them too. The database with raw data is available from the authors (excluding the correspondence and contact information of editors in order to protect the anonymity of responses).

### Statistical analysis

Data were presented as frequencies for categorical variables, and, depending on the distribution of data, means or medians with 95% confidence intervals (CI) for continuous variables. Differences between citation counts of original and duplicate publications were tested by Wilcoxon test, while the equal distributions of inclusion of authors’ responses within the published notices was tested with a chi-squared test. All analyses were conducted using MedCalc v.12.5 (MedCalc, Ostend, Belgium). All calculations were done on DP cases for which MEDLINE assigned the DP tag to at least one article. This means that we did not include 6 DP notices incorrectly tagged as DPs, as our goal was to follow up on papers tagged by NLM and the analysis of notices as sources of DPs would go beyond the scope of this research, and had been previously reported (*8*).

## Results

On 16 January 2013, there were 1011 citations indexed (with a DP [Publication Type] tag) in MEDLINE amounting to 555 possible cases of different DPs (as MEDLINE indexes both the original and duplicate(s) article(s) with the DP tags). Following the analysis of published notices, communication with editors and comparison of full text of citations, we were able to analyse 549 cases (for 6 we were unable to retrieve full texts or receive a response from editors).

Out of 549 analysed cases, 359 (65%) were cases of DP while 190 (35%) were cases incorrectly indexed as duplicates. The majority of the duplicates (N = 200; 56%) occurred due to the publishers’ actions, most commonly publication of the same article in two different issues of the same journal, while those occurring due to authors’ actions (N = 159; 44%) occurred most commonly due to submission of the same manuscript to two different journals (Table 1).

**Table 1:** Reasons for duplicate publications indexed in MEDLINE

**Duplications due to**	**Duplicate publications, N (%)**		
	**With published notice**	**Without published notice**	**Total**
**Author’s actions:**	120 (62)	39 (24)	159 (44)
Submission to multiple journals	75 (39)	34 (21)	109 (30)
Study fragmentation	30 (15)	3 (2)	33 (9)
Submission without co-author(s) approval	12 (6)	0 (0)	12 (3)
Plagiarism	1 (1)	2 (1)	3 (1)
Pharmaceutical company sent the same database to two different teams for write-up	1 (1)	0 (0)	1 (0)
Authors lost communication with the journal following prolonged article processing	1 (1)	0 (0)	1 (0)
**Publisher’s actions:**	74 (38)	126 (76)	200 (56)
Article published twice in different volumes	62 (32)	45 (27)	107 (30)
Double publication in sister journals or agreement between the journals without citing the original	2 (1)	62 (38)	64 (18)
Article published twice in the same volume	6 (3)	15 (9)	21 (6)
Wrong indexation sent to MEDLINE	0 (0)	4 (2)	4 (1)
Journal’s oversight of authors declaration of secondary submission/redaction error	4 (2)	0 (0)	4 (1)
Total	194 (54)	165 (46)	359 (100)

Cases incorrectly indexed were most commonly intentional republications, and until 4 June 2018 NLM had corrected 138 (73%) of them (Table 2).

**Table 2:** Cases incorrectly indexed as duplicate publications in MEDLINE

**Type of article**	**Articles, N (%)**	**Articles corrected by NLM until 24 June 2018, N (%)**
Republished or translated articles citing the original^†^	51 (27)	34 (67)
Simultaneous publications (*e.g.* guidelines, editorials)	50 (26)	36 (72)
Articles with no obvious reasons for DP indexation^‡^	30 (16)	30 (100)
Similar papers (*e.g.* study fragmentation, expanded reviews)	17 (9)	4 (23)
Articles from the *International Journal of Biostatistics*	12 (6)	12 (100)
Notices of duplicate publication^†^	9 (5)	9 (100)
Article updates^†^	8 (4)	6 (75)
Articles on controlled ecological life support system	7 (4)	7 (100)
Articles with supplements indexed separately	4 (2)	0 (0)
Letter to editor that prevents duplication	1 (1)	0 (0)
Comment and a reply to the comment	1 (1)	0 (0)
Total	190 (100)	138 (73)
^†^NLM Fact Sheet specifically states that article reprints, updates and notices of duplication are not indexed as DPs, but have their own separate indexation (*10*). ^‡^After searching MEDLINE using authors’ names and title keywords we found no similar articles. NLM removed the DP tag after we contacted them. NLM - National Library of Medicine. DP - duplicate publications.		

Duplicate publications tags were not visible in default PubMed search result output. This information was visible only after expanding the “Supplemental information” field (Supplementary material Figures S1-2). Additionally, of the 181 (72%) of 250 cases for which we got the response from the editors, only 1 (1%) editor confirmed being aware of the DP tagging in MEDLINE, 166 (92%) did not explicitly confirm or deny it, but opened an investigation of the issue after we had contacted them, and one (1%) asked to acknowledge us for alerting them of the DP. Fifteen editors (8%) explicitly stated they were not aware of the practice.

Out of 359 cases of DPs, 185 (51%) had a published notice (included is a case of triplicate duplication, even though the notice only addresses the duplication). Almost two thirds of published notices (117 of 185, 63%, χ^2^ = 12.454, df = 1, P < 0.001) reported author’s actions as the reason for the duplication (Table 1). Of the 185 notices, 174 (94%) were visible in PubMed and designated most commonly as comments (N = 103; 56%) or errata (N = 54; 29%) (Table 3).

**Table 3:** Type of comments/corrections visible in MEDLINE to alert users about duplicate publications

**Comment or Correction type**	**Retracted DP, N (%)**	**Non-retracted DP, N (%)**	**Total, N (%)**
Comment in	3 (12)	102 (64)	105 (56)
Erratum in	5 (20)	49 (31)	54 (29)
Retraction in	13 (52)	0 (0)	13 (7)
Comment on	0 (0)	1 (1)	1 (1)
Corrected and republished from	0 (0)	1 (1)	1 (1)
Not visible in MEDLINE*	4 (16)	7 (4)	11 (6)
Total	25 (14)	160 (86)	185 (100)
*The published notice was either visible on the journal’s website or we were informed by the editor of its existence, but the notice was not linked to the article in MEDLINE. DP - duplicate publications.			

Notices were visible (clearly linked to the article) on the article’s website for only 48 (26%) DPs, while for 4 (2%) they were visible as part of PubMed Central (PMC) records. In 11 (6%) cases journals did not have the article indexed online, while for the rest (N = 122; 66%) the notices were not visible. The titles of published notices varied in their format of which the most common was a general notification (N = 119; 64%) (Table 4).

**Table 4:** Headings of notifications publishers’ use to alert readers of duplicate publications

**Heading type**	**Retracted DP, N (%)**	**Non-retracted DP, N (%)**	**Total, N (%)**
General notification:	15 (60)	104 (65)	119 (64)
A statement of retractions	3 (12)	0 (0)	3 (2)
Correction(s)	1 (4)	10 (6)	11 (6)
Correction: Notice of redundant publication	0 (0)	4 (3)	4 (2)
Corrigendum	0 (0)	2 (1)	2 (1)
Department of error	0 (0)	1 (1)	1 (1)
Duplicate/dual publication	0 (0)	13 (8)	13 (7)
Duplicate submission and publication of correspondence	0 (0)	1 (1)	1 (1)
Editorial	0 (0)	2 (1)	2 (1)
Editor(-s/-ial) or publisher’s note/announcement/comment/statement	1 (4)	7 (4)	8 (4)
Erratum(-a)	2 (8)	16 (10)	18 (10)
Erratum: (double publication/notice of redundant publication)	0 (0)	2 (1)	2 (1)
Letter to the editors	0 (0)	2 (1)	2 (1)
Notice (to readers)	0 (0)	4 (3)	4 (2)
Notice of (inadvertent) duplicate/dual publication	1 (4)	29 (18)	30 (16)
Notice of redundant publication	1 (4)	8 (5)	9 (5)
Retraction (notice/ of publication)	5 (20)	0 (0)	5 (3)
Scientific publications: ethical issues or fraud	0 (0)	1 (1)	1 (1)
Statement of duplicate publication	0 (0)	1 (1)	1 (1)
Table of contents notification	0 (0)	1 (1)	1 (1)
Table of contents: Retraction	1 (4)	0 (0)	1 (1)
Notification with the full title of the DP (*e.g.* “Notice of duplicate submission: Use of the superficial femoral vein as a replacement for large veins”)	6 (24)	15 (9)	23 (12)
Only the full title of the DP	0 (0)	16 (9)	13 (7)
Notification with the DP title and reference (*e.g.* “Notice of duplicate publication: The use of mitomycin-C for respiratory papillomas. Clinical, histologic and biochemical correlation. Saudi Med J 2005;26:1737-45”)	4 (16)	9 (6)	13 (7)
DP reference only (*e.g.* “Transfusion 1998;38:79-85”)	0 (0)	8 (5)	8 (4)
Notification with the DP reference (*e.g.* “Notice of Redundant Publication: Am J Kidney Dis. 2010;55:639-47”)	0 (0)	8 (5)	8 (4)
DP title and reference only (*e.g.* “Exercise induced anaphylaxis and pregnancy. Anaesthesia 2007;62:420.”)	0 (0)	1 (1)	1 (1)
Total	25 (100)	160 (100)	185 (100)
The published notice was either visible on the journal’s website or we were informed by the editor of its existence, but the notice was not linked to the article in MEDLINE. DP - duplicate publications.			

The full text of the notices was mostly freely available (N = 141; 76%). For 29 (16%) it was restricted behind pay-walls, while for 9 (5%) the notice, and for 6 (3%) the whole volume containing the notice, were not available online. Half of the notices that cited authors’ errors as the reasons for duplication (66 out of 117, 56%, χ^2^ = 1.675, df = 1, P = 0.196) included a statement or acknowledgment from the authors.

Only 25 (7%) of 359 DP cases were retracted. Of the 25, 13 (52%) had a “Retraction in” designation in MEDLINE, 5 (20%) were marked as having errata, 3 (12%) as having comments, and 4 (16%) had no visible notice (Table 3). On the journals’ webpages, 10 articles (40%) were marked as retracted, 12 (48%) had no visible notification, and for 3 (12%) the journal’s webpage did not have the volume online. Half of the retractions occurred due to author’s (N = 13; 52%) and half due to publisher’s actions (N = 12; 48%).

Following our contact with the editors, additional 9 notices were published: 8 retractions, of which 6 for publishers’ errors and 2 for authors’ errors, and 1 notice of redundant publication (author’s error). Furthermore, 3 online versions of articles were updated to include a statement that an article was a peer reviewed version of a manuscript submitted to a conference. For additional 38 cases, editors said they would publish a notice, but have not done so even after a year, and for 11 cases editors specified they would not publish a notice (Supplementary material Table S1).

The earliest DP was published in 1980, and the first retracted in 1990. On average, there were 11 DPs *per* year (95% CI: 7 - 14), with a peak of 28 in 2004 (Supplementary material Figure S3-4). Overall median time from the original to duplicate publication was 3 months (95% CI: 2 - 3), and from the DP to the notice of duplication (or retraction) 8 months (95% CI: 6 - 10). The median number of total citations for original and duplicate articles was 6 (95% CI: 5 - 7); and the median average citation by year 0.5 (95% CI: 0.4 - 0.6). Hundred and ten (16%) of the 691 articles indexed as DP were never cited (the total of 691 articles is based on counting all original, duplicate and triplicate citations indexed and having a DP tag). There were no differences between the total citation count and the average citation *per* year between the duplicates and their corresponding original articles (P = 0.125 and P = 0.438, respectively; Supplementary material Table S2). Separate analysis of duplicates with published notices also yielded no differences between their total citation counts (P = 0.106), average citation *per* year (P = 0.259) or citations that occurred two years following publication of the duplication notice (P = 0.835) (Supplementary material Table S2). In cases of duplications that occurred due to the publisher’s actions (N = 200), the number of authors and the by-line order of DPs and original articles were in 93% (N = 185) of the cases identical; while for DPs occurring due to author’s actions (N = 159) they were identical in 43% (N = 68) of the cases (Supplementary material Table S3).

## Discussion

Our study demonstrated that, despite the existence of clear guidelines on how to deal with duplicate publications, almost half of the DPs (46%) tagged in MEDLINE have not been addressed by journals and only 9% were adequately addressed, *i.e.* rectracted (*1*, *6*, *7*).

While our study was not designed to look at the reasons for the lack of adherence to editorial policies, previous studies have reported on the unwillingness and difficulties in addressing misconduct and errors (*e.g.* contact ambiguity, (in)appropriate timeliness, inability to obtain raw data), and the inconsistencies of implementing misconduct policies in biomedical journals, and scientific communities (*14*, *15*, *17*-*20*). It is also possible that DPs do not invoke the same reaction as claims of fabricated or falsified data. In our study, we did not receive any response from the journals for 28% (69 of 250) of enquiries. Additionally, the 38 editors who said they would address the DPs have not done so within a year of being contacted.

The limitation of our study was the fact that we communicated with listed contact persons of the journals, so we may have not reached some of the editors, as was previously reported by researchers handling scientific misconduct (*16*). Furthermore, our sample was based only on articles tagged as duplicates by the NLM indexing staff, and they most likely represent only a portion of all DP cases in MEDLINE as 6 of 9 notices that were incorrectly tagged as DPs in our sample mentioned additional cases of DPs, and a study of 2343 retracted articles in MEDLINE which found 506 retractions were due to DPs, had only 16 of publications overlapping with ours (*8*, *21*). We tried contacting the authors of the study to compare our datasets and determine the combined number of DPs, but received no replies. As many journals still do not routinely check article similarity upon submission, and those that do so are often unable to compare them to all existing publications due to differences in indexing and coverage of most common similarity check services, the number of DPs could be even larger.

Our study also showed that, similar to retracted publications, DPs continued to be cited even after journals published notices of duplication (*22*-*25*). The likely reasons behind this are the following: 1) the DP indexation is not easily visible (example in S1 Figures 1-3); 2) editors use a wide range of notices to alert users of DPs (Table 4); 3) papers are often cited based only on read abstracts or taken from lists of references of other authors; 4) publishers’ website are rarely used to access articles (where the notice of DP may exist); and 5) current reference management software (*e.g.* EndNote, Zotero) do not alert users of subsequent notices, errata and retractions, which leaves researchers unaware of changes following publication of a paper (*26*-*29*).

Based on the negative impact duplicate publications have on meta-analyses, the time spent on excluding duplicates when conducting systematic reviews, and problems in correcting duplicates as demonstrated in our study; bibliographical databases, journals and publishers should increase their efforts to clearly mark retracted and duplicate publications (perhaps by adding an additional field alongside the title of DP, such as Web of Science practice for retracted publications), as well as to clearly identify the final versions of papers (*4*, *5*, *30*-*32*). Furthermore, as has already been suggested, tools could be developed to alert users of changes to papers they previously cited (*24*).

Finally, as indexing in bibliographic databases can result in errors, (35% incorrectly indexed articles in our study), as the online versions are often not updated (only 24% of notices were visible on the journal’s website in our study), and as there are currently no legislative ways to ensure proper handling of corrections or versioning or articles; it is perhaps time to consider partial or complete collaborative volunteering of the public and researchers, or use of machine learning algorithms, for tagging and classifying of scientific records (*33*-*38*). Additionally, scientific publishing should perhaps adopt to a more ‘living’ and user-friendly versions of articles in which editing is made easier after the original publication, *e.g.* Wiki content pages (and content management system) or Clinicaltrials.gov archive site records, which showcase updated versions, but keep a record of all changes that occurred (*36*, *39*). Such ‘living’ and interactive articles could also solve the problem of the invisibility and discrepancy in the usage and titles of notices, errata, comments, corrigenda, reprints and retractions, as reported previously and confirmed in our study (*34*, *35*, *40*-*42*).

The two main categories we used to classify DPs, the authors’ and the publishers’ actions, invoke a question whether publishers’ actions should be retracted. Unlike author’s actions, they rarely contain changes in the author by-line order of two publications, nor otherwise indicate deliberate actions so a simple erratum that would add the designation of a reprint, or an updated version of the article could remedy the situation (*21*, *40*). As authors’ actions leading to DPs constitute detrimental research practice, a clear distinction between the two DP types should be made.

Our study has also shown that the number of articles tagged as DPs in MEDLINE has been decreasing in the last several years, which may reflect a positive change in the publishing culture, where a submission of a same article to more than one journal is now widely considered unacceptable and wasteful. However, with no visible transparency of how many indexers detect or report DPs, and clear indication from NLM that they do not regularly check for duplications, the DPs tagged in MEDLINE may not reflect the actual number of biomedical DPs.

Our finding that almost a half of DPs remain unaddressed by journals and that many editors have failed to address the DPs in a reasonable time after we informed them of their existence, raise a question should these (in)actions be considered editorial misconduct and who should policy them or alert the readers or authors of such fallacies. Furthermore, what should be done for DPs that may be discovered in the future when more and more of old journal issues will be retroactively indexed in bibliographic databases? Should a moratorium be placed on scientific misconduct, as some suggest, or should duplicates be addressed by COPE, editorial organizations or bibliographic databases themselves, even when the journals in question are no longer publishing or the authors can no longer be contacted (*43*). After all, while investigating scientific misconduct requires a lot of time and effort, a comparison of two (or more) similar published records is a more straightforward task.

In conclusion, taking into consideration the current situation with DP indexing and the digital tools available, there is a need for concrete actions of stakeholders to ensure that duplicate publications are identified, mapped and the reasons behind the duplications resolved in a prompt and transparent way across all scientific fields.

## Supplementary material

Supplementary figures and tables
