# Supplementary material for: Correcting duplicate publications: follow up study of MEDLINE tagged duplications
Source: Biochem Med (Zagreb). 2018 Dec 15;29(1):010201. doi: 10.11613/BM.2019.010201 (PMC6294161; doi:10.11613/BM.2019.010201)
Supplement: Supplementary file 1 — Supplementary figures and tables [file bm-29-1-010201-S1.pdf]

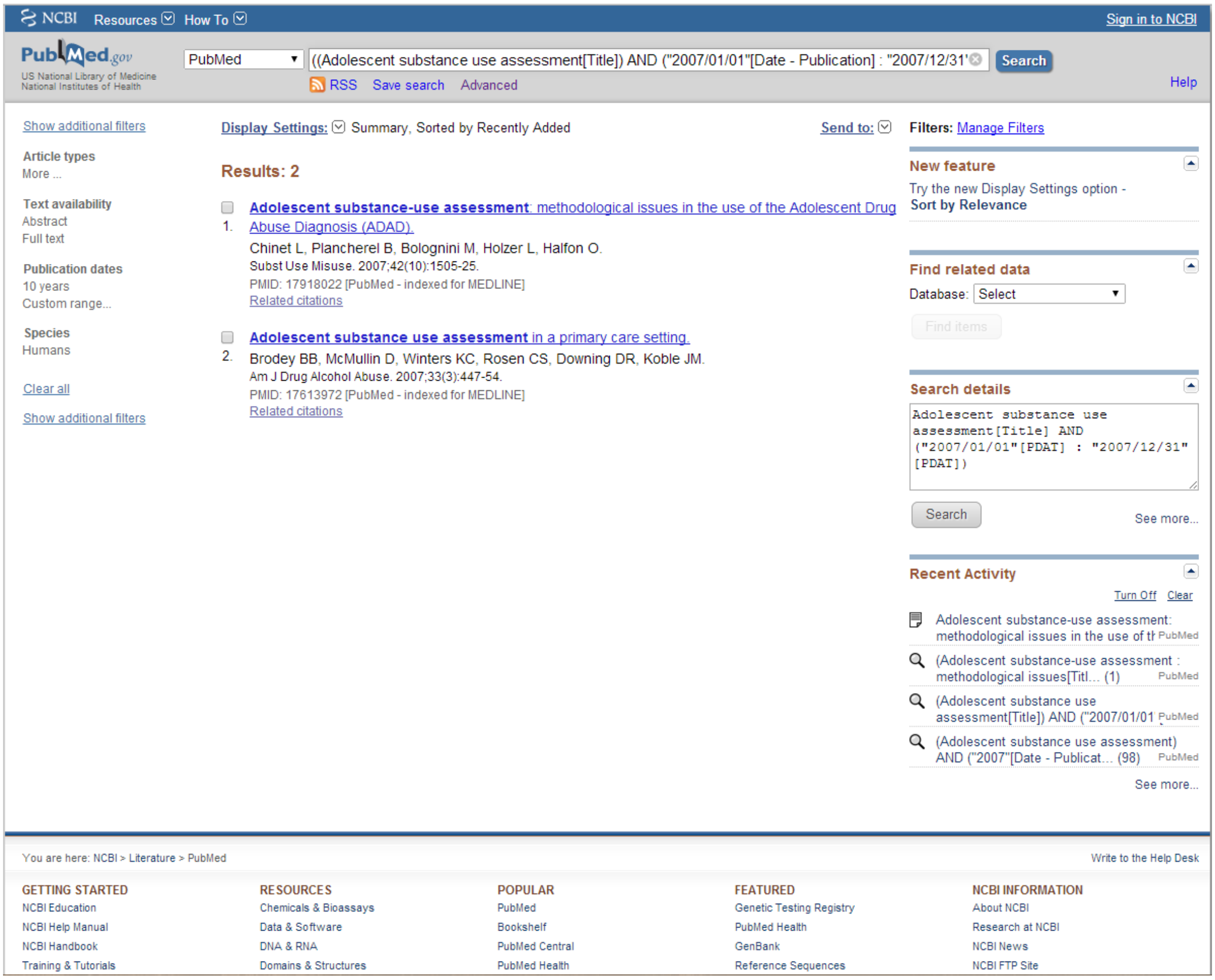

Figure S1. Default search results view in PubMed. The example shows results of search for articles with titles containing the phrase: "adolescent substance use" published in 2007. The first article is a duplicate publication but this is not indicated anywhere on the screen. (This figure is a screenshot of National Library of Medicine Web page, available in the public domain.)

(A)

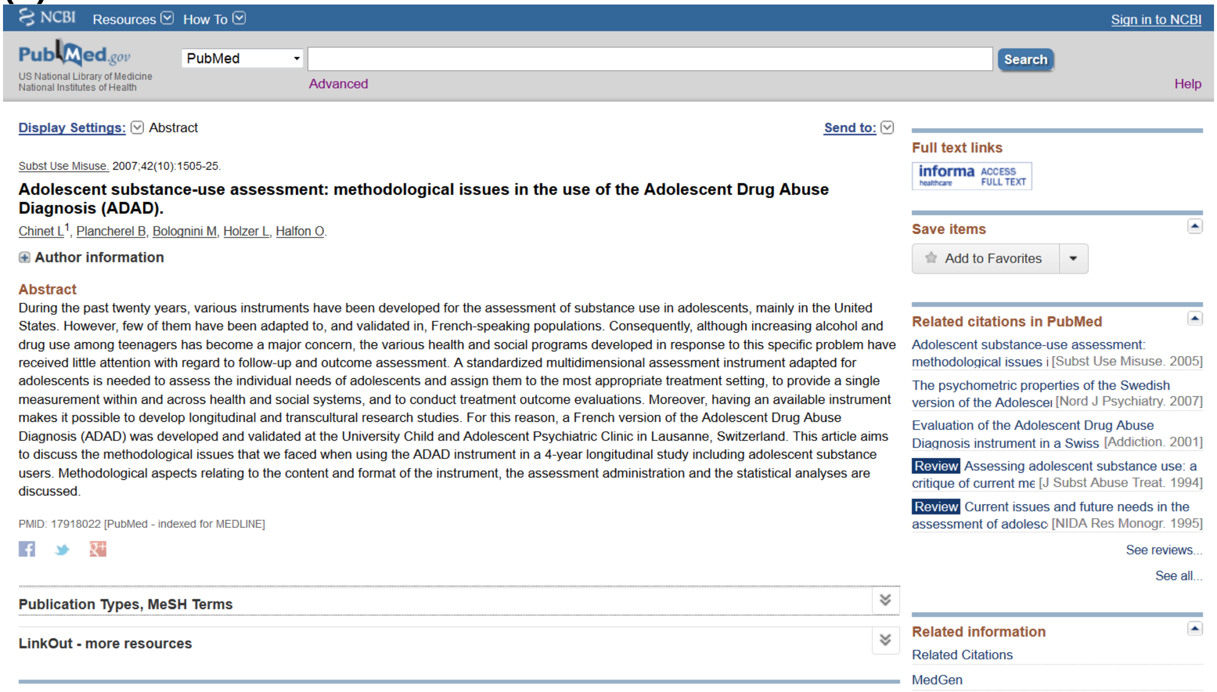
NCBI Resources How To Sign in to NCBI

PubMed.gov US National Library of Medicine National Institutes of Health

PubMed Search Advanced Help

Display Settings: Abstract Send to:

Subst Use Misuse. 2007;42(10):1505-25.

Adolescent substance-use assessment: methodological issues in the use of the Adolescent Drug Abuse Diagnosis (ADAD).

Chinet L[1], Plancherel B, Bolognini M, Holzer L, Halfon O.

Author information

Abstract

During the past twenty years, various instruments have been developed for the assessment of substance use in adolescents, mainly in the United States. However, few of them have been adapted to, and validated in, French-speaking populations. Consequently, although increasing alcohol and drug use among teenagers has become a major concern, the various health and social programs developed in response to this specific problem have received little attention with regard to follow-up and outcome assessment. A standardized multidimensional assessment instrument adapted for adolescents is needed to assess the individual needs of adolescents and assign them to the most appropriate treatment setting, to provide a single measurement within and across health and social systems, and to conduct treatment outcome evaluations. Moreover, having an available instrument makes it possible to develop longitudinal and transcultural research studies. For this reason, a French version of the Adolescent Drug Abuse Diagnosis (ADAD) was developed and validated at the University Child and Adolescent Psychiatric Clinic in Lausanne, Switzerland. This article aims to discuss the methodological issues that we faced when using the ADAD instrument in a 4-year longitudinal study including adolescent substance users. Methodological aspects relating to the content and format of the instrument, the assessment administration and the statistical analyses are discussed.

PMID: 17918022 [PubMed - indexed for MEDLINE]

Publication Types, MeSH Terms

LinkOut - more resources

Full text links

informa healthcare ACCESS FULL TEXT

Save items

Add to Favorites

Related citations in PubMed

Adolescent substance-use assessment: methodological issues i [Subst Use Misuse. 2005]

The psychometric properties of the Swedish version of the Adolescei [Nord J Psychiatry. 2007]

Evaluation of the Adolescent Drug Abuse Diagnosis instrument in a Swiss [Addiction. 2001]

Review Assessing adolescent substance use: a critique of current me [J Subst Abuse Treat. 1994]

Review Current issues and future needs in the assessment of adolesc [NIDA Res Monogr. 1995]

See reviews...

See all...

Related information

Related Citations

MedGen

(B)

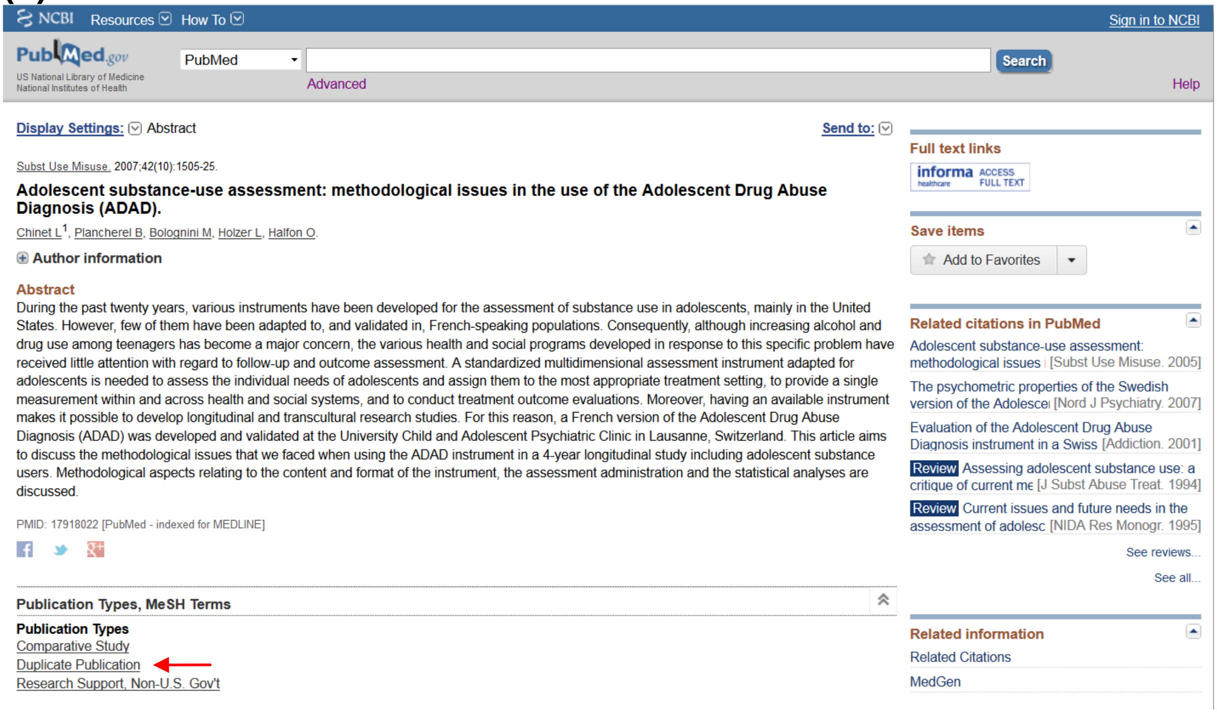
NCBI Resources How To Sign in to NCBI

PubMed.gov US National Library of Medicine National Institutes of Health

PubMed Search Advanced Help

Display Settings: Abstract Send to:

Subst Use Misuse. 2007;42(10):1505-25.

Adolescent substance-use assessment: methodological issues in the use of the Adolescent Drug Abuse Diagnosis (ADAD).

Chinet L[1], Plancherel B, Bolognini M, Holzer L, Halfon O.

Author information

Abstract

During the past twenty years, various instruments have been developed for the assessment of substance use in adolescents, mainly in the United States. However, few of them have been adapted to, and validated in, French-speaking populations. Consequently, although increasing alcohol and drug use among teenagers has become a major concern, the various health and social programs developed in response to this specific problem have received little attention with regard to follow-up and outcome assessment. A standardized multidimensional assessment instrument adapted for adolescents is needed to assess the individual needs of adolescents and assign them to the most appropriate treatment setting, to provide a single measurement within and across health and social systems, and to conduct treatment outcome evaluations. Moreover, having an available instrument makes it possible to develop longitudinal and transcultural research studies. For this reason, a French version of the Adolescent Drug Abuse Diagnosis (ADAD) was developed and validated at the University Child and Adolescent Psychiatric Clinic in Lausanne, Switzerland. This article aims to discuss the methodological issues that we faced when using the ADAD instrument in a 4-year longitudinal study including adolescent substance users. Methodological aspects relating to the content and format of the instrument, the assessment administration and the statistical analyses are discussed.

PMID: 17918022 [PubMed - indexed for MEDLINE]

Publication Types, MeSH Terms

Publication Types

Comparative Study

Duplicate Publication

Research Support, Non-U.S. Gov't

Full text links

informa healthcare ACCESS FULL TEXT

Save items

Add to Favorites

Related citations in PubMed

Adolescent substance-use assessment: methodological issues i [Subst Use Misuse. 2005]

The psychometric properties of the Swedish version of the Adolescei [Nord J Psychiatry. 2007]

Evaluation of the Adolescent Drug Abuse Diagnosis instrument in a Swiss [Addiction. 2001]

Review Assessing adolescent substance use: a critique of current me [J Subst Abuse Treat. 1994]

Review Current issues and future needs in the assessment of adolesc [NIDA Res Monogr. 1995]

See reviews...

See all...

Related information

Related Citations

MedGen

Figure S2. Default PubMed abstract view of a duplicate publication presented in Figure S1. The information that this article was tagged by NLM as a duplicate publication is not indicated on the default abstract screen (A), but only upon expanding the supplementary information field – "Publication Types, MeSH terms". (B) These figures are screenshots of National Library of Medicine Web pages, available in the public domain.

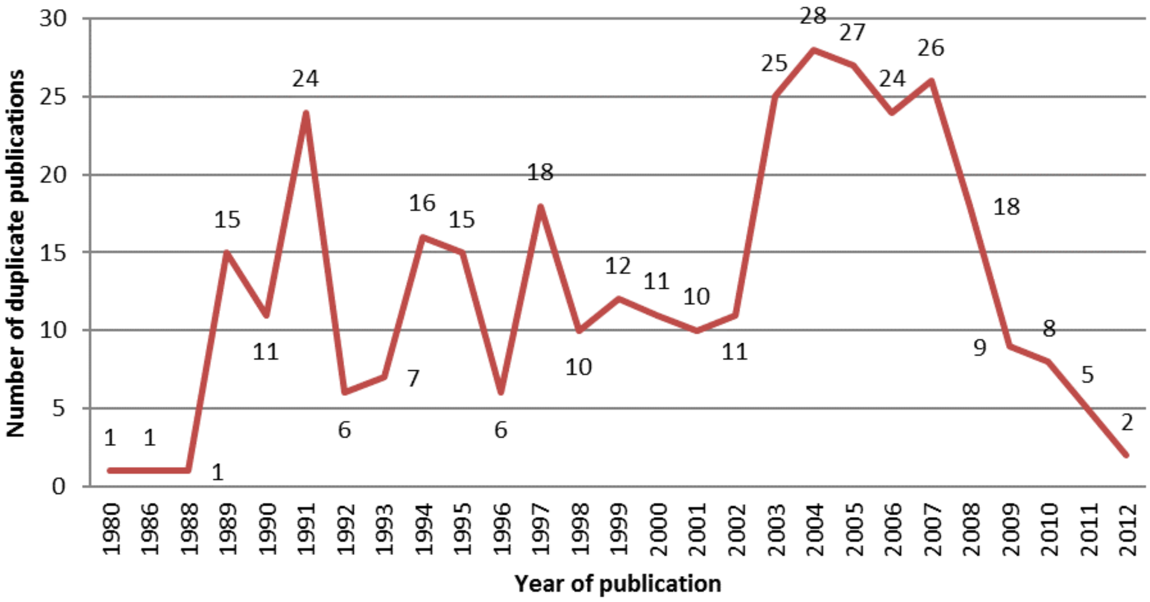

Figure S3. Number of unique instances of duplicate publications per year in Medline (N = 347; 12 triplicate publications are excluded).

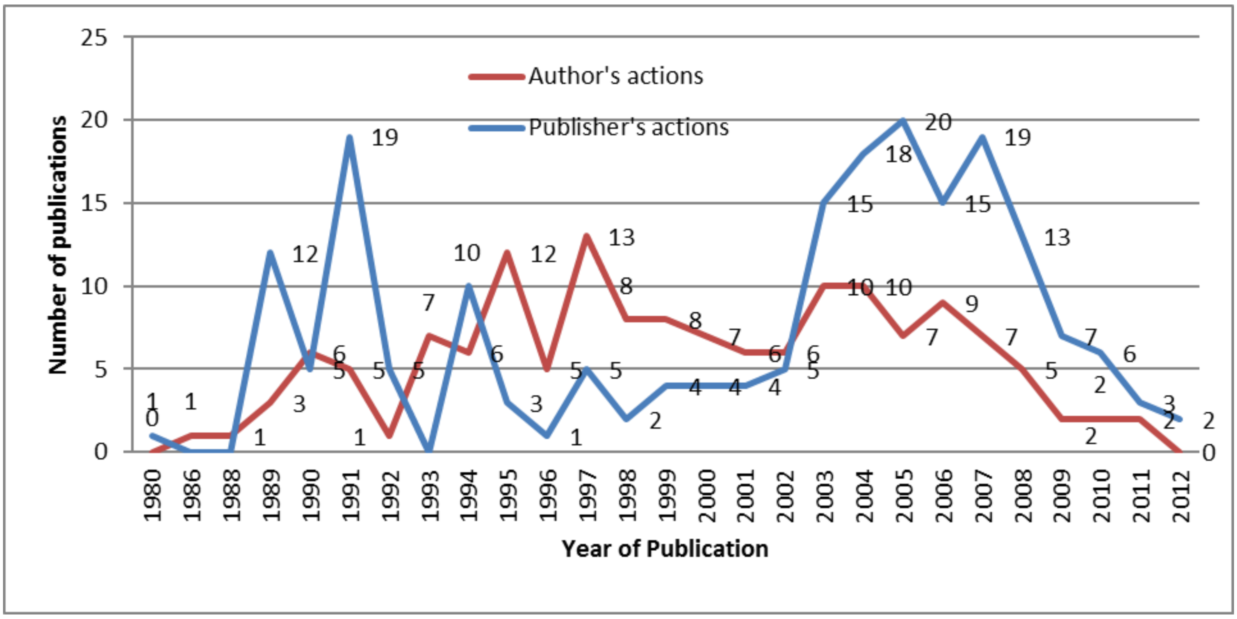

Figure S4. Number of unique instances of duplicate publications per year in Medline (N = 347; 12 triplicate publications are excluded) grouped according to the reason of duplication (author’s or publisher’s actions).

Table S1. Replies of editors regarding duplicate publications indexed in MEDLINE

Reply	N (%)
This is not a case of duplicate publication	62 (34)
Will publish notice/follow COPE guidelines*	44 (24)
Will investigate	28 (16)
Asked about advice on how to handle the issue or remove the duplication	12 (7)
Will not publish the notice	11 (6)
It was a common practice then to publish proceedings and later the paper	3
Cannot investigate the issue in detail as it was long time ago	2
This occurred in time of the previous editor	1
It’s a translated article, only missing is that statement	1
The publisher intentionally publishing articles in two sister journals	1
As it is marked as duplicate there is no need to retract it	1
The editors republished the article in a special issue intentionally	1
The authors apologised and the NLM had been informed	1
Did not specify the course of action	10 (6)
Notified about already existing notice	7 (4)
The other journal should investigate due to a later publication date	5 (3)
Retracted without informing us of the investigation results	2 (1)

COPE - Committee on Publication Ethics. *6 out of the 44 had published a notice till May 2017

Table S2. Citation counts of duplicate publications indexed in MEDLINE

Characteristic	Original articles (N = 309)†	Duplicate articles (N = 309)†	P*
Total citation count (Md, 95% CI)	7 (5 - 8)	6 (5 - 7)	0.125
Average Citation by year (Md, 95% CI)	0.6 (0.4 - 0.7)	0.6 (0.4 - 0.7)	0.438
	**Articles with published notices of DP (N = 145)‡**	**Duplicates with published notices of DP (N = 145)‡**	
Total citation count (Md, 95% CI)	12 (7-15)	10 (7 - 13)	0.444
Average Citation by year (Md, 95% CI)	0.9 (0.6 - 1.1)	0.8 (0.7 - 1.1)	0.828
Total citation count two years following publication of a notice (Md, 95% CI)§	6 (4 - 9)	6 (5 - 8)	0.835

Md – median. CI – confidence interval. DP – duplicate publication. *Wilcoxon signed-rank test. †12 cases of triplicate publications are excluded, as well as 38 indexed articles without matching duplicate publication. ‡Of 185 cases of DP with published notices, 31 had no matching tagged duplicate publication, and 9 referred to triplicate publications. §N = 143 for citations to articles two years following the notice of duplication, as 2 notices were confirmed as published by the editors but due to them belonging to old issues their full text or date of publication could not be retrieved by the journals' editors.

Table S3. Authorship by-line changes from original to duplicate publication

Reason behind duplication	Identical by-line order, N (%)	Different by-line order, N (%)	Different number of authors, N (%)	Different author team*, N (%)	Number of authors listed, N (%)	Total, N (%)
**Authors' action**	68 (43)	18 (11)	57 (36)	16 (10)	0 (0)	159 (44)
Submission to multiple journals	56 (22)	10 (45)	35 (52)	8 (50)	0 (0)	109 (30)
Study fragmentation	9 (4)	7 (32)	13 (19)	4 (25)	0 (0)	33 (9)
Submission without co-author(s) approval	2 (1)	1 (5)	9 (13)	0 (0)	0 (0)	12 (3)
Plagiarism	0 (0)	0 (0)	0 (0)	3 (19)	0 (0)	3 (1)
Pharmaceutical company sent the same database to two different teams for write-up	0 (0)	0 (0)	0 (0)	1 (6)	0 (0)	1 (0)
Authors lost communication with the journal following prolonged article processing	1 (0)	0 (0)	0 (0)	0 (0)	0 (0)	1 (0)
**Publisher's action**	185 (93)	4 (2)	10 (5)	0 (0)	1 (1)	200 (56)
Article published twice in different volumes	105 (42)	1 (5)	1 (1)	0 (0)	0 (0)	107 (30)
Double publication in sister journals or agreement between journals without citing the original	54 (21)	3 (14)	6 (9)	0 (0)	1 (100)	64 (18)
Article published twice in the same volume	20 (8)	0 (0)	1 (1)	0 (0)	0 (0)	21 (6)
Wrong indexation sent to MEDLINE	4 (2)	0 (0)	0 (0)	0 (0)	0 (0)	4 (1)
Journal's oversight of authors declaration of secondary submission/redaction error	2 (1)	0 (0)	2 (3)	0 (0)	0 (0)	4 (1)
Total	253 (70)	22 (6)	67 (19)	16 (4)	1 (0)	359 (100)

*These refer to the same number of authors in the by-line, but different identity of at least one of the authors.

Template Letter to Editors

Dear Editor,
The following article(s) published in your journal(s) have been marked as duplicate publications in MEDLINE (If you click on publication types below the abstract you will see the duplicate designation):
(Links specific for each case of DP was inserted here)
As a part of our research on duplicate publications (see our presentation at the Peer Review conference at http://www.peerreviewcongress.org/abstracts_2013.html#16), we were unable to find any acknowledgment or published notice from your journal(s) about this duplication, and were wondering whether you were aware that these articles were designated as duplicate in MEDLINE? As part of our follow up research, we would be grateful if you would contact us about this issue, and let us know if you as an editor have been aware of the NLM practice of tagging highly similar publications as duplicate publications irrespective of official notice by the journal(s).
We are also in constant contact with the NLM indexers about duplicate issues, and are happy to work with you to resolve this matter, if you find that the duplicate indexation is incorrect and should be removed.
Thank you for considering this request.
We look forward to your comments and suggestions.

Kind regards,
Mario Malicki, MD, MA
Department of Research in Biomedicine and Health
University of Split School of Medicine
Šoltanska 2
21000 Split
Croatia
Phone: +385 21 557 820
Fax: +385 21 557 820
e-mail: mario.malicki@mefst.hr
ORCID ID: http://orcid.org/0000-0003-0698-1930
